# Selective Activation of hTRPV1 by *N*-Geranyl Cyclopropylcarboxamide, an Amiloride-Insensitive Salt Taste Enhancer

**DOI:** 10.1371/journal.pone.0089062

**Published:** 2014-02-20

**Authors:** Min Jung Kim, Hee Jin Son, Yiseul Kim, Hae-Jin Kweon, Byung-Chang Suh, Vijay Lyall, Mee-Ra Rhyu

**Affiliations:** 1 Division of Metabolism and Functionality Research, Korea Food Research Institute, Bundang-gu, Sungnam-si, Gyeonggi-do, Republic of Korea; 2 Department of Brain Science, DaeguGyeongbuk Institute of Science and Technology (DGIST), Daegu, Republic of Korea; 3 Department of Physiology and Biophysics, Virginia Commonwealth University, Richmond, Virginia, United States of America; University of Tokyo, Japan

## Abstract

TRPV1t, a variant of the transient receptor potential vanilloid-1 (TRPV1) has been proposed as a constitutively active, non-selective cation channel as a putative amiloride-insensitive salt taste receptor and shares many properties with TRPV1. Based on our previous chorda tympani taste nerve recordings in rodents and human sensory evaluations, we proposed that *N*-geranylcyclopropylcarboxamide (NGCC), a novel synthetic compound, acts as a salt taste enhancer by modulating the amiloride/benzamil-insensitive Na^+^ entry pathways. As an extension of this work, we investigated NGCC-induced human TRPV1 (hTRPV1) activation using a Ca^2+^-flux signaling assay in cultured cells. NGCC enhanced Ca^2+^ influx in hTRPV1-expressing cells in a dose-dependent manner (EC_50_ = 115 µM). NGCC-induced Ca^2+^ influx was significantly attenuated by ruthenium red (RR; 30 µM), a non-specific blocker of TRP channels and capsazepine (CZP; 5 µM), a specific antagonist of TRPV1, implying that NGCC directly activates hTRPV1. TRPA1 is often co-expressed with TRPV1 in sensory neurons. Therefore, we also investigated the effects of NGCC on hTRPA1-expressing cells. Similar to hTRPV1, NGCC enhanced Ca^2+^ influx in hTRPA1-expressing cells (EC_50_ = 83.65 µM). The NGCC-induced Ca^2+^ influx in hTRPA1-expressing cells was blocked by RR (30 µM) and HC-030031 (100 µM), a specific antagonist of TRPA1. These results suggested that NGCC selectively activates TRPV1 and TRPA1 in cultured cells. These data may provide additional support for our previous hypothesis that NGCC interacts with TRPV1 variant cation channel, a putative amiloride/benzamil-insensitive salt taste pathway in the anterior taste receptive field.

## Introduction

Sodium ion (Na^+^) is the principal extracellular ion and is essential for maintaining homeostasis in the body. At concentrations below 250 mM Na^+^ is generally appetitive and helps to enhance the flavor intensity of food. People in developed countries ingest far more salt than is required to maintain a normal Na^+^ balance. In the United States, Na^+^ intakes in adults are around 3900 mg per day which is significantly greater than the maximum recommended value of 2300 mg per day. Approximately 77% of the Na^+^ consumed is derived from processed foods in USA [1]. The excessive salt consumption is associated with many diseases, including hypertension, heart attack, stroke, fluid retention, weight gain, Ca^2+^ deficiency and osteoporosis, stomach cancer [2]–[5]. Therefore, it is imperative that the food industry should make a considerable effort in finding novel ways to reduce salt content in their food products and shift their focus to search for salt taste enhancers as one of the alternative approaches to lower salt intake in the general population [6].

Currently, there is evidence for the presence of at least two salt taste receptors in the tongue which are involved in appetitive salt taste perception that demonstrate different sensitivity to amiloride and benzamil (Bz). One salt taste receptor is the amiloride-sensitive epithelial Na^+^ channel (ENaC), the Na^+^ specific salt taste receptor in fungiform papilla taste receptor cells (TRCs) in the anterior tongue in mammals, including humans [7]–[10]. In rats and mice, ENaC consists of three subunits: α, β, and γ. In humans, an additional subunit, δ is expressed, so the human TRC ENaC is additionally composed of δ, β and γ subunits. ENaC is blocked by amiloride and Bz, however, the δβγ ENaC (IC_50_ around 2.7 µM) is 10 times less sensitive to amiloride than the human αβγ ENaC (IC_50_ around 0.11 µM) [11]. However, amiloride at 100 µM (approximately 50 times greater than the IC_50_ for human δβγ ENaC) reduced only 5–40% of lingual surface potential to salt stimuli in humans measured by gustometer, while amiloride inhibited about 70% of chorda tympani (CT) taste nerve response to NaCl [12], [13]. These results suggest that in both humans and some rodents, besides ENaC, additional taste receptors must be involved in salt taste transduction.

Single unit recordings of the CT nerve [14], [15] suggest that additional salt taste transduction pathway must exist which respond to a variety of cations, including Na^+^, K^+^, NH_4_^+^ and Ca^2+^ that are amiloride-insensitive. In some mice the magnitude of the amiloride-insensitive component is genetically determined [10], [16]. Lyall *et al.* first presented electrophysiological evidence that modulators of the transient receptor potential vanilloid-1 channel (TRPV1), such as resiniferatoxin (RTX) and capsaicin, modulate the amiloride- and Bz-insensitive NaCl CT response in a biphasic manner, at low concentrations enhancing and high concentrations inhibiting the Bz-insensitive NaCl CT response in WT mice and rats [17], [18]. The constitutive Bz-insensitive NaCl CT response and the enhanced response in the presence of RTX and capsaicin were inhibited by SB-366791, a specific TRPV1 blocker. In addition, other TRPV1 modulators, such as ethanol, nicotine, temperature, and adenosine triphosphate (ATP) were shown to modulate the Bz-insensitive NaCl CT response. These data suggested that TRPV1-dependent pathway may be a major component of the Bz-insensitive NaCl CT response in Sprague-Dawley rats and WT mice. In addition, the SB-366791-sensitive spontaneous Bz-insensitive NaCl CT response in the absence of TRPV1 modulators, at room temperature, close to neutral pH suggests that a variant of TRPV1 (TRPV1t) may be involved in amiloride-insensitive salt taste [14], [17], [19], [21]. Although studies performed by Smith et al. (2012) did not support this hypothesis in mice [22], a correlation between genetic variation in TRPV1 and salt taste perception in human was observed by human salt taste perception studies [23]. The rs8065080 polymorphism from T allele to C allele in TRPV1 gene significantly increased suprathreshold salt taste sensitivity in human. These results provide additional support for a link between TRPV1t and salt taste in humans. At high concentration NaCl is aversive (>500 mM) and is sensed by two aversive pathways localized in bitter and sour sensing TRCs [24].

Among monovalent chloride salts, choline chloride is suggested to be a salt taste enhancer and replacer for Na^+^ because choline chloride has salt taste-enhancing properties in animal model [25]. Accordingly, several choline-containing compounds were synthesized to develop as salt substitutes and/or enhancers [26]. Based on this report, *N*-geranylcyclopropylcarboxamide (NGCC), *N*-geranylisobutanamide, *N*-geranyl 2-methylbutanamide, allyl *N*-geranylcarbamate, and *N*-cyclopropyl E2,Z6-nonadienamide were synthesized by International Flavors & Fragrances (IFF). The effects of these compounds on the amiloride-sensitive and amiloride-insensitive salt taste pathways havebeen investigated in WT and TRPV1 KO mice by electrophysiological studies. Of these compounds, only NGCC produced biphasic effects on the amiloride-insensitive salt taste pathway, without altering the amiloride-sensitive salt taste pathway [27]. Most importantly, NGCC at the concentrations around which it maximally enhanced the Bz-insensitive NaCl CT response in rodents also enhanced salt taste perception of NaCl solutions (60–80 mM) in human subjects.

Since NGCC enhances salt taste responses on both rodents and humans, in this paper we performed Ca^2+^-flux signaling assay in hTRPV1-transfected Human Embryonic Kidney (HEK293T) cells to investigate whether NGCC directly activates hTRPV1, and hence, the putative amiloride-insensitive salt taste receptor, TRPV1t. To test whether NGCC specifically activates TRPV1 we also tested its effects on the αβγ human ENaC (hENaC)-expressing HEK293T cells by membrane potential assay. The effects of NGCC on acid-sensing ion channels 1a (ASIC1a) were also investigated because the proton-gated ASIC is amiloride-insensitive and belongs to the ENaC/Degenerin superfamily. In addition, we also investigated the effect of NGCC on hTRPA1-transfected HEK293T cells because TRPA1 and TRPV1 are frequently co-expressed in sensory neurons.

## Materials and Methods

### Materials

**Figure 1:**
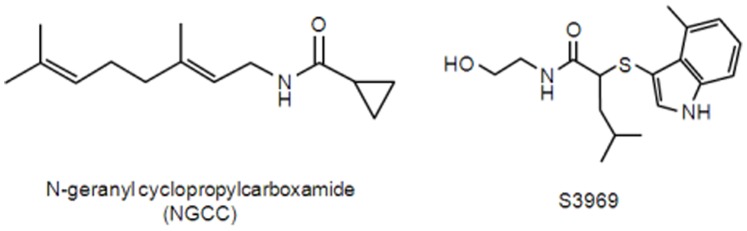
Chemical structure of S3969 and NGCC.

Capsaicin, capsazepine (CPZ), ruthenium red (RR), HC-030031, and benzamil (Bz) were purchased from Sigma-Aldrich (St. Louis, MO, USA). Allylisothiocyanate (AITC) was obtained from Wako Pure Chemicals Industries Ltd. (Osaka, Japan). S3969 was synthesized as described previously [11] by professor Jeon at Kwangwoon University. NGCC was kindly provided by Dr. Mark Dewis at IFF (Union Beach, NJ, USA). S3969 and NGCC structures are shown in Figure 1. All media used in cell cultures were obtained from Life Technologies, Inc. (Grand Island, NY, USA).

### Cell Culture and Transfection

hTRPV1 was constructed by amplification of the hTRPV1 region (NCBI accession number: NG_029716.1) and cloned into pEAK10 vector (Edge Biosystems, Gaithersburg, MD, USA). The hTRPV1 construct was amplified by PCR and the nucleotide sequence of the hTRPV1 gene was confirmed by sequencing with an ABI 3130 DNA genetic analyzer (Applied Biosystems, Foster City, CA, USA). The HEK293T cells which were cultured at 37°C in Dulbecco’s Modified Eagle’s Medium (DMEM) supplemented with 10% fetal bovine serum (FBS) and 1%penicillin/streptomycin were seeded onto a 100-mm dish and transfected with the hTRPV1 expression plasmid using Lipofectamine 2000 (Invitrogen). After 6 h, transfected cells were seeded onto 96-well black-wall plate (BD Falcon Labware, Franklin Lakes, NJ, USA) for 18–26 h prior to their use in an experiment.

The α, β, and γ hENaC subunits were cloned from OriGene (Rockville, MD; GenBank accession number NM_00001038 for *SCNN1A*, NM_000336 for *SCNN1B* and NM_001039 for *SCNN1G*). α, GFP-tagged β, and γ hENaC were cloned into a CMV promoter-based vector and expressed constitutively. The HEK293T cells were seeded onto a 35 mm dish and transfected with α, β, and γ hENaC expression plasmid using Lipofectamine 2000 (Invitrogen). Then, transfected cells were seeded onto 96-well black-wall plate for 18–26 h prior to their use in an experiment.

Mouse ASIC1a (mASIC1a) was a kind gift from John A. Wemmie (University of Iowa, IA, USA). For electrophysiological experiments, HEK293T cells were cultured in DMEM supplemented with 10% FBS and 0.2% penicillin/streptomycin and transiently transfected using Lipofectamine 2000 (Invitrogen) with various cDNAs. For TRPV1 channel expression, cells were transfected with hTRPV1, for ASIC1a homomeric channel expression, cells were transfected with mASIC1a. When needed, 0.1–0.2 µg of cDNA encoding tetrameric red fluorescence protein (DsRed) was co-transfected with the cDNA as a marker for successfully transfected cells. For non-transfected cells, cells were transfected with 0.1–0.2 µg of cDNA encoding DsRed alone. The next day, cells were plated onto poly-L-lysine-coated coverslip chips, and fluorescent cells were studied within 1–2 days.

Flp-In 293 cell lines stably expressing hTRPA1constructed as previously reported [21] was a gift from the professor Takumi Misaka at University of Tokyo. The hTRPA1-expressing cells were cultured in DMEM containing 10% FBS and 0.02% hygromycin B (Invitrogen). Flp-In 293 cell lines were maintained in DMEM containing 10% FBS. All cells were incubated at 37°C in a humidified atmosphere of 5% CO_2_. Cultured hTRPA1-expressing cells and Flp-In 293 cell lines were seeded onto 96-well black-wall plate for 24 h and used for Ca^2+^ responses to AITC and NGCC.

### Ca^2+^ Imaging Analysis of the Cellular Response of hTRPV1- or hTRPA1-expressing Cells

Mock-transfected HEK293T cells, hTRPV1-expressing HEK293T cells, non-hTRPA1-expressing Flp-In 293 cells, and hTRPA1-expressing Flp-In 293 cells grown in 96-well black-wall plates were rinsed with assay buffer (130 mM NaCl, 10 mM glucose, 5 mM KCl, 2 mMCaCl_2_, 1.2 mM MgCl_2_, and 10 mM HEPES, pH 7.4) and loaded with 5 µM Fura-2 AM (Invitrogen) for 30 min at 27°C. The cells were washed with assay buffer and treated with ligands. AITC (10 µM) and capsaicin (1 µM) dissolved in assay buffer were used as the hTRPA1 and hTRPV1 ligands, respectively. hTRPA1 and hTRPV1 expressing cells were treated with 30 µM NGCC dissolved in assay buffer. The fluorescence intensities of Fura-2 excited at 340 and 380 nm were simultaneously measured at 510 nm using a computer-controlled filter changer (Lambda DG4; Sutter, San Rafael, CA, USA), an Andor Luca CCD camera (Andor Technology, Belfast, UK), and an inverted fluorescence microscope (IX-71; Olympus, Tokyo, Japan). Intracellular calcium images were recorded every 3-s for 60-s and analyzed using MetaFluor software (Molecular Devices, Sunnyvale, CA, USA). For the blocking assay, 30 µM RR or 1 µM CPZ was added with capsaicin or NGCC in hTRPV1-expressing cells and 30 µM RR or 100 µM HC-030031 was added with AITC or NGCC in hTRPA1-expressing cells.

### Measurement of Ca^2+^ Influx in hTRPV1- or hTRPA1-expressing Cells

The ligand-induced changes on cytosolic Ca^2+^ level were monitored by a FlexStation III microplate reader (Molecular Devices). Fluo-4 AM (5 µM, Molecular Probes, Eugene, OR, USA) in assay buffer was loaded to the cells in 96-well black-wall plates for 30 min at 27°C. After dye treatment, each chemical was added to each well at 17 s and changes on intracellular Ca^2+^ level were monitored by relative fluorescence units (ΔRFU, Ex = 485 nm; Em = 516 nm) for 120 s. The chemicals including capsaicin (1×10^−4^−3×10 µM) and NGCC (1×10^−4^−1×10^3^ µM) were treated to hTRPV1-expressing cells and those including AITC (1×10^−4^−3×10^2^ µM) and NGCC (1×10^−4^−1×10^3^ µM) were treated to hTRPA1-expressing cells. The responses from at least three wells receiving the same stimulus were averaged. Plots of amplitude versus concentration were fitted using the Hill equation. For the blocking assay, 30 µM RR or 1 µM CPZ was added with capsaicin or NGCC in hTRPV1-expressing cells and 30 µM RR or 100 µM HC-030031 was added with AITC or NGCC in hTRPA1-expressing cells.

### Membrane Potential Assay on αβγ hENaC-expressing Cells

hENaC-expressing cells were characterized using FLIPER Membrane Potential (FMP) Assay Kit (Molecular Devices Corporation, Sunnyvale, CA, USA). In this assay, changes in the membrane potential were quantified via fluorescence change caused by FMP dye. The hENaC-expressing cells were washed with assay buffer (containing: 130 mM NaCl, 10 mM glucose, 5 mM KCl, 2 mM CaCl_2_, 1.2 mM MgCl_2_, and 10 mM HEPES, pH adjusted to 7.4 with NaOH) and subsequently, FMP blue-dye in assay buffer was loaded into the cells at room temperature for 30 min. After dye treatment, S3969 (0.03−10 µM), an ENaC agonist, S3969+Bz (ENaC antagonist, 0.01 µM), or NGCC (0.01−10 µM) was treated to hENaC-expressing cells. The plate was assayed in a FlexStation III plate reader (Molecular devices Corporation) by excitation at 530 nm and measuring the emission at 560 nm.

### Whole-cell Patch Clamp Recording

The whole-cell configuration was used to voltage-clamp at room temperature (22–25°C). Electrodes pulled from glass micropipette capillaries (Sutter Instrument) had resistances of 2–2.5 MΩ, and series resistance errors were compensated >60%. Fast and slow capacitances were compensated before the application of test-pulse. Recordings were performed using aHEKAEPC-10 amplifier with pulse software (HEKA Elektronik). The pipette solution contained: 140 mM KCl, 5 mM MgCl_2_, 10 mM HEPES, 0.1 mM 1,2-bis(2-aminophenoxy) ethane *N,N,N’,N’*-tetraacetic acid(BAPTA), 3 mM Na_2_ATP, and 0.1 mM Na_3_GTP, adjusted to pH 7.4 with KOH. The external Ringer’s solution used for recording TRPV1 and TRPA1 currents contained: 150 mM NaCl, 5 mM KCl, 1 mM MgCl_2_, 2 mM EGTA, 10 mM Glucose, and 10 mM HEPES, adjusted to pH 7.4 with NaOH. TRPV1 and TRPA1 currents were recorded by holding the cell at −80 mV. The external Ringer’s solution used for recording ASIC current contained: 160 mM NaCl, 5 mM KCl, 1 mM MgCl_2_, 2 mM CaCl_2_, and 10 mM HEPES, adjusted to pH 7.4 with tetramethylammonium hydroxide. For acidic solution of pH 6.0, HEPES was replaced with MES. ASIC currents were recorded by holding the cell at −70 mV. Reagents were obtained as follows: BAPTA, Na_2_ATP, Na_3_GTP, EGTA, and tetramethylammonium hydroxide (Sigma), HEPES (Calbiochem), MES (Alfa Aesar), and other chemicals (Merck).

### Statistical Analysis

The statistical significance of the results was examined through one-way analysis of variance (ANOVA) using Duncan’s multiple range test. Dose–response analyses were carried out with GraphPad Prism software (GraphPad Software Inc., San Diego, CA, USA). Data are presented as means ± SEM.

## Results

### NGCC-induced Activation of TRPV1t/TRPV1

**Figure 2:**
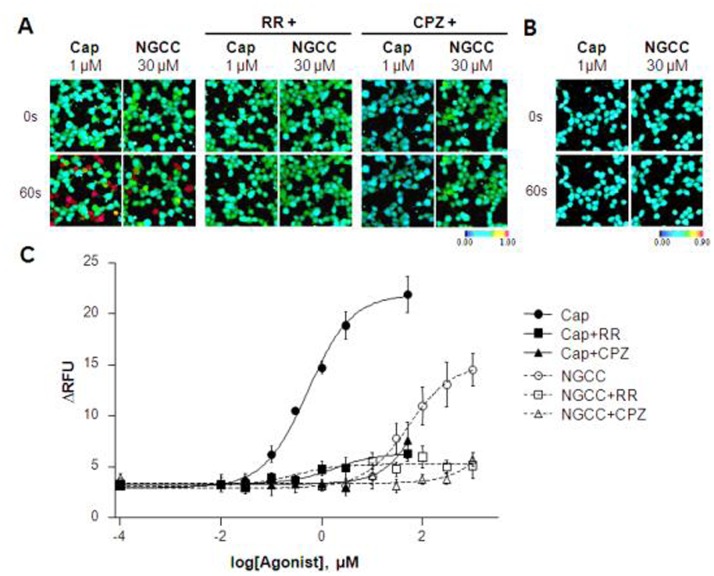
Calcium responses in hTRPV1-expressing cells stimulated with capsaicin (Cap) and NGCC. (**A**) Cells expressing hTRPV1 were loaded with Fura-2 AM and Ca^2+^ images were obtained at 0 and 60 s after stimulation with Cap or NGCC. The non-specific blocker of TRP channels, RR (30 µM), and the specific TRPV1 antagonist, CPZ (5 µM), were added to test the selectivity of Cap and NGCC. Representative ratiometric images are shown after treatment with Cap and NGCC. (**B**) As a control, the Ca^2+^ response was monitored in non-hTRPV1-expressing HEK 293T cells treated with Cap or NGCC. (**C**) The effects of Cap or NGCC treatment were quantified using Calcium-4 in a cell-based assay in the presence or absence of 30 µM RR or 5 µM CPZ. Experiments were repeated in triplicate and data points represent the means ± SEM (n = 3).

The effects of capsaicin and NGCC on hTRPV1-expressing cells or mock-transfected cells are shown in Figure 2A and Figure 2B at the single concentration. Capsaicin, a specific agonist of hTRPV1, was used as a positive control. As a result, NGCC (30 µM) increased Ca^2+^ influx in hTRPV1-expressing cells, and RR (30 µM) and CPZ (1 µM) blocked NGCC activity to baseline levels (Figure 2A). NGCC induced no changes in fluorescence in mock-transfected cells (Figure 2B). NGCC produced a concentration-dependent increase in the intracellular Ca^2+^ in hTRPV1-expressing cells (Figure 2C). The EC_50_ values for capsaicin and NGCC on hTRPV1-expressing cells were 0.527 µM and 115 µM, respectively. CPZ (1 µM) blocked the effects of capsaicin and NGCC, however, it did not completely inhibit the hTRPV1 activity at high concentrations of capsaicin (≥30 µM) and NGCC (≥10^3^ µM). RR (30 µM) also inhibited capsaicin- and NGCC-induced hTRPV1 activation, but was less effective than CPZ (1 µM). In the presence of RR (30 µM) no increase in intracellular Ca^2+^ was observed at low concentrations of the two agonists (both less than 1 µM).

**Figure 3:**
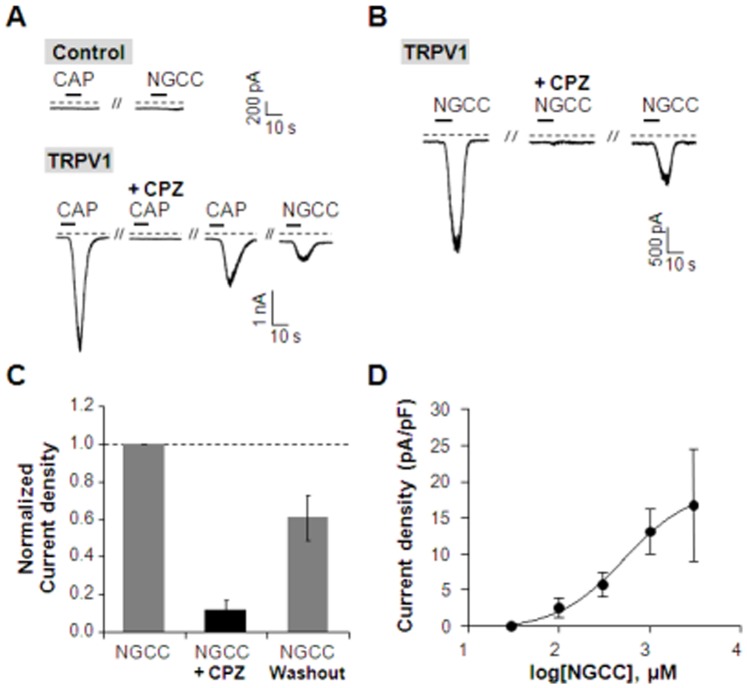
Activation of TRPV1 by capsaicin and NGCC. (**A**) TRPV1 current was activated by capsaicin (100 nM) and reversibly blocked by TRPV1 antagonist, CPZ (1 µM) in HEK293T cells transiently transfected with hTRPV1. NGCC (1 mM) induced an inward current in a capsaicin-sensitive cell. Neither capsaicin nor NGCC evoked the currents in non-transfected cells (n = 3). Dashed lines indicate zero current. (**B**) NGCC-induced currents were blocked by CPZ in hTRPV1-expressing HEK293T cells. (**C**) Summary of normalized current density in cells expressing hTRPV1 (n = 4). (**D**) Current density of the first pulse was normalized to 1.0. NGCC triggered inward currents in a dose-dependent manner in hTRPV1-expressing HEK293T cells (n = 3). Results are presented as the mean ± SEM.

To verify whether hTRPV1 was activated by NGCC, we patch-clamped mock-transfected or hTRPV1-expressing cells (Figure 3). We observed the effect of NGCC on cells transiently expressing hTRPV1. As a positive control, we used capsaicin (100 nM) to induce TRPV1 currents. Bath application of capsaicin for 10 seconds induced an inward current in the cell expressing hTRPV1, but not in the non-transfected cell (Figure 3A). Capsaicin-induced current was blocked by preincubation of the cell with Ringer’s solution containing CPZ (1 µM) for 20 seconds before the second application of capsaicin and the response recovered after wash out of CPZ. Moreover, NGCC (10^3^ µM) also triggered an inward current in the same cell. Like capsaicin, NGCC triggered no currents in non-transfected cells. NGCC-induced currents were also inhibited by CPZ, and then, partially recovered after wash out of CPZ similar to capsaicin-evoked currents in cells expressing hTRPV1 (Figure 3B). Normalized current density was decreased to 0.12±0.05 (n = 4) by treatment of CPZ and recovered to 0.61±0.12 (n = 4) after wash out of CPZ (Figure 3C). NGCC activated inward currents in cells expressing hTRPV1 in a concentration-dependent manner (Figure 3D). These results suggest that NGCC is an agonist for TRPV1.

### Effects of NGCC on Amiloride/Benzamil-sensitive Salt Taste Receptor, ENaC

**Figure 4:**
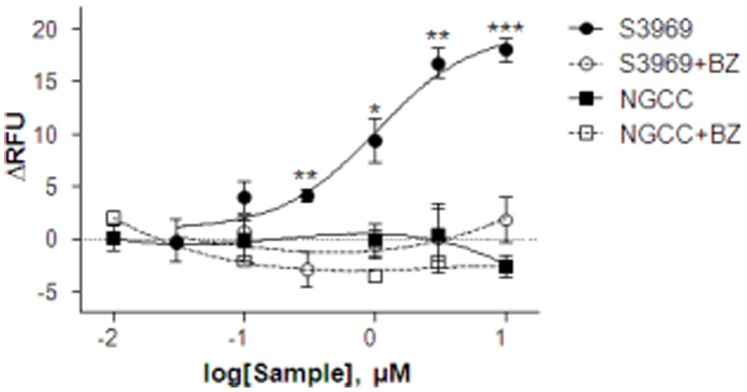
Effects of S3969 or NGCC on αβγ hENaC-expressing cells. Cells expressing αβγ-hENaC were loaded with FMP blue-dye and the effects of S3969 or NGCC were quantitatively evaluated using membrane potential assay. S3969 depolarized membrane potential on αβγ hENaC-expressing cells and benzamil (Bz) effectively inhibited S3969 activity. NGCC showed no effect on αβγhENaC-expressing cells. Experiments were repeated in triplicate and data points represent the means ± SEM (n = 3–4).

Prior to investigating the effects of NGCC on the amiloride-sensitive salt taste receptor, the efficiency of αβγ hENaC-expressing cells was evaluated using S3969 (Figure 4). Between 0.03 and 10 µM, S3969 depolarized the membrane potential in hENaC expressing cells in a concentration-dependent manner with an EC_50_ value of 1.262±0.290 µM. No effect of S3969 was observed in the continuous presence of Bz. Following this, we investigated the effect of NGCC on αβγ hENaC-expressing cells using same system. Although we tested a wide range of NGCC concentrations, no significant changes in membrane potential were observed in αβγ hENaC-expressing cells relative to cells stimulated by buffer alone. Thus, similar to our earlier findings [27], NGCC did not affect amiloride/benzamil-sensitive salt taste receptor.

### Effects of NGCC on Amiloride-sensitive Acid-sensing Ion Channel, ASIC

**Figure 5:**
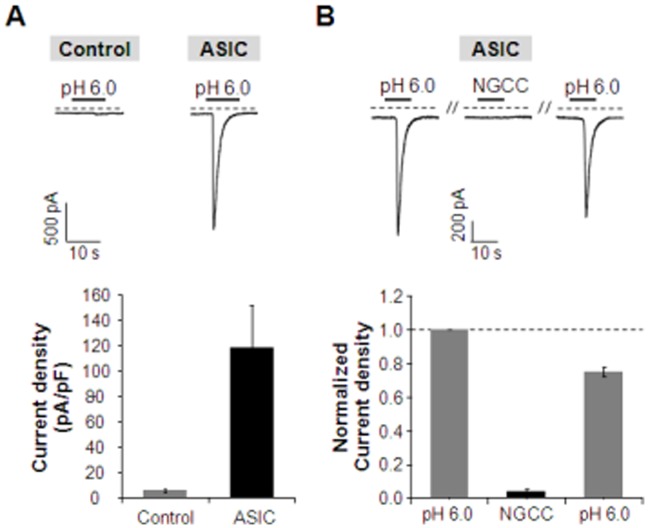
Effects of NGCC in amiloride-sensitive ASIC1a. (**A**) Rapid change of extracellular pH from 7.4 to 6.0 for 10 seconds induced an inward current in HEK293T cells transiently transfected with mASIC1a. Current density of pH 6.0-evoked inward currents is 5.52±1.33 pA/pF (n = 3) and 118.1±33.0 pA/pF (n = 3) in non-transfected cells and mASIC1a-expressing cells, respectively. (**B**) mASIC1a currents were triggered by extracellular pH drop from 7.4 to 6.0. However, ASIC1a channels are not sensitive to NGCC (1 mM). The time interval between each stimulation is 120 seconds. Normalized current density was measured in cells expressing mASIC1a (n = 5). Results are presented as the mean ± SEM.

To determine whether NGCC also activates amiloride-sensitive acid-sensing ion channels (ASICs) which belong to ENaC/DEG superfamily of ion channels [28], we also tested the effect of NGCC on ASIC1a homomeric channels in HEK293T cells. Previous study showed that ASIC1 subunit is expressed in taste receptor cells and involved in taste transduction [29], [30]. Rapid change of extracellular pH from 7.4 to 6.0 for 10 seconds evoked inward currents in cells expressing homomeric ASIC1a channels (Figure 5A). Although HEK293 cells are reported to express endogenous human ASIC1a [31], the current density of pH 6.0-induced currents in non-transfected HEK293T cells was 5.52±1.33 pA/pF (n = 3), while the current density of pH 6.0-induced currents in mASIC1a-expressing HEK293T cells was 118.1±33.0 pA/pF (n = 3). Therefore, we regarded pH 6.0-induced currents as mASIC1a-mediated currents in cells transiently expressing mASIC1a. For the purpose of this study, we applied pH 6.0 solution or NGCC on the extracellular site of cells expressing mASIC1a to determine whether NGCC has potential for activating ASIC1a homomeric channels. As a result, mASIC1a-mediated currents were activated by extracellular pH change from 7.4 to 6.0, but not by application of NGCC (Figure 5B).

### Activation of hTRPA1 by NGCC

**Figure 6:**
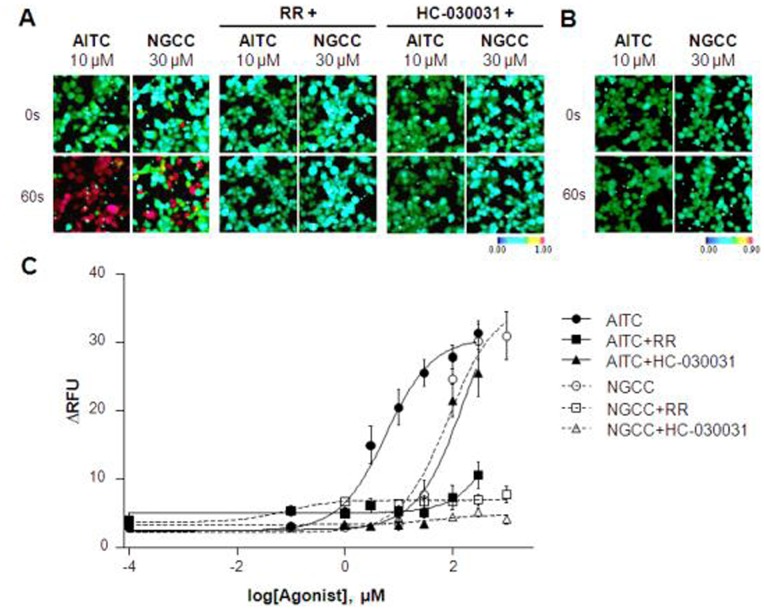
Calcium responses in hTRPA1-expressing cells stimulated with AITC and NGCC. (**A**) hTRPA1-expressing cells were loaded with Fura-2 AM and Ca^2+^ images were obtained at 0 and 60 s after stimulation with AITC or NGCC. The selectivity of AITC and NGCC was tested by adding the non-specific blocker of TRP channels, RR (30 µM), or a specific TRPA1 antagonist, HC-030031 (100 µM). Representative ratiometric images are shown after treatment with AITC and NGCC. (**B**) As a control, the Ca^2+^ response to AITC or NGCC treatment was monitored in mock-transfected Flp-In 293 cells. (**C**) AITC and NGCC treatment showed dose-dependent effects in hTRPA1-expressing cells. The effects of AITC or NGCC treatment in the presence or absence of 30 µM RR or 100 µM HC-030031 were quantified using Calcium-4 in a cell-based assay. Experiments were repeated in triplicate and data points represent the means ± SEM (n = 3).

Finally, the effects of AITC and NGCC on hTRPA1-expressing cells were investigated in a single dose. AITC, a TRPA1 agonist was used as a positive control. Figure 6A showed that the intracellular Ca^2+^ concentration was increased after 10 µM AITC or 30 µM NGCC treatment and the Ca^2+^ influx induced by 10 µM AITC and 30 µM NGCC was significantly inhibited by RR (30 µM) and HC-030031 (100 µM). Comparison of NGCC activity in non-hTRPA1-expressing Flp-In 293 cells showed that NGCC reacts specifically with hTRPA1 (Figure 6B). Subsequently, AITC- and NGCC-induced Ca^2+^ influx was monitored in a concentration-dependent manner (Figure 6C). As a result, AITC and NGCC activated Ca^2+^ influx in hTRPA1-expressing cells in a concentration-dependent manner. The EC_50_ values for AITC and NGCC on hTRPA1-expressing cells were 5.852 µM and 83.65 µM, respectively. RR (30 µM) and HC-030031 (100 µM) almost blocked NGCC activity on hTRPA1-expressing cells. However, RR (30 µM) and HC-030031 (100 µM) partially blocked AITC activity below 30 µM and 10 µM, respectively.

**Figure 7:**
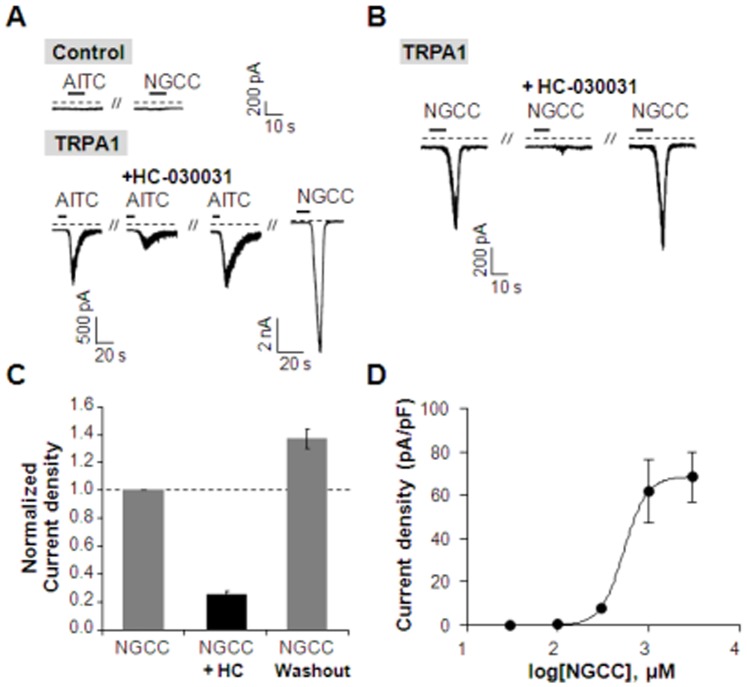
Activation of hTRPA1 by AITC and NGCC. (**A**) AITC (100 µM) induced an inward current in hTRPA1-expressing cells. AITC-induced currents were partially inhibited by TRPA1 antagonist, HC-030031 (100 µM) and recovered after wash out. NGCC triggered an inward current in a AITC-sensitive cell. Neither AITC nor NGCC evoked the currents in non-transfected cells (n = 3). (**B**) NGCC-induced currents were blocked by HC-030031 in hTRPA1-expressing cells. (**C**) Summary of normalized current density in cells expressing hTRPA1 (n = 4). (**D**) NGCC triggered inward currents in a dose-dependent manner in hTRPA1-expressing cells (n = 4). Results are presented as the mean ± SEM.

These results were also confirmed using patch clamp technique. We tested the effect of NGCC on cells stably expressing hTRPA1. Application of AITC (100 µM) which is well-known as an agonist of TRPA1 for 10 seconds, evoked an inward current in the hTRPA1-expressing cell, but not in the non-transfected cell (Figure 7A). AITC-induced current was partially blocked by preincubating the cells with Ringer’s solution containing HC-030031 (100 µM) for 30 seconds before the second application of AITC and the response recovered after wash out of HC-030031. In the same cell, NGCC (10^3^ µM) also induced an inward current. Consistent with Ca^2+^ imaging data, the amplitude of current induced by NGCC (10^3^ µM) was bigger than that of AITC (100 µM)-induced current. However, NGCC triggered no current in non-transfected cells as like AITC. NGCC-induced currents were almost completely inhibited by HC-030031 and the response recovered after wash out of HC-030031 in hTRPA1-expressing cells (Figure 7B). Normalized current density was decreased to 0.25±0.03 (n = 4) by addition of HC-030031 and recovered to 1.37±0.07 (n = 4) (Figure 7C). NGCC triggered inward currents in cells stably expressing hTRPA1 in a dose-dependent manner (Figure 7D).

## Discussion

The sense of taste relies on Na^+^ for sensing salty taste. Because excessive salt consumption is associated with numerous diseases, there is a great incentive to develop Na^+^ substitutes and salt taste enhancers that can help in reducing salt intake. Previously, we have shown that NGCC is a salt taste modifier. Specifically, using whole CT nerve recordings in WT and TRPV1 KO mice and stimulating the tongue with NaCl+Bz (an ENaC inhibitor), NGCC produced biphasic effects on the Bz-insensitive NaCl CT response. At low concentrations NGCC enhanced and at high concentrations inhibited the Bz-insensitive NaCl CT response [27]. In addition, at the concentrations at which NGCC produced the maximum increase in the CT response in rodents, it produced a synergistic salt taste-enhancing effect in human psychophysical tests. At the concentrations that NGCC inhibited the Bz-insensitive NaCl CT response in rodents, it produced a salt masking effect in human psychophysical tests. NGCC did not enhance the Bz-sensitive NaCl CT response [27]. In our earlier paper we did not have direct evidence that NGCC modulates the activity of TRPV1 and ENaC is insensitive to it. In this paper we provide direct evidence that NGCC modulates the activity of hTRPV1 by monitoring NGCC-induced change in intracellular Ca^2+^ levels in hTRPV1-expressing cells.

As shown in Fig. 2, capsaicin, a TRPV1 agonist, produced a dose dependent increase in [Ca^2+^]_i_ in HEK 293 cells expressing hTRPV1, the maximum increase in [Ca^2+^]_i_ was observed at around 100 µM capsaicin. The EC_50_ for capsaicin was around 0.5 µM. In our rat CT recordings, capsaicin produced a biphasic effect on the Bz-insensitive NaCl CT response [17]. The maximum increase in the NaCl+Bz CT response was obtained at 40 µM capsaicin. At concentrations >40 µM capsaicin inhibited the NaCl+Bz CT response and at 200 µM capsaicin completely inhibited the NaCl+Bz CT response to the rinse baseline level. Relative to capsaicin, NGCC produced a significantly smaller increase in [Ca^2+^]_i_ and the dose-response relationship was shifted to the right to higher agonist concentration. The EC_50_ for NGCC was around 115 µM. In contrast to the effects of NGCC on the expressed hTRPV1, in our earlier human sensory studies NGCC produced a significant enhancement in NaCl taste at 5 and 10 µM [27]. These studies suggest that the threshold concentration of NGCC for producing enhancement in the NaCl taste perception is significantly lower than the concentration that produces pain.

It is important to note that unlike our studies with expressed hTRPV1 (Figure 2), in taste cells NGCC [27] and capsaicin [17], ethanol [18], nicotine [32], NGCC [27] and naturally occurring glycol-conjugated peptides [33] produced biphasic effects on the Bz-insensitive neural responses and in human salt taste sensory evaluation studies [27], [34]. The biphasic effects of the above TRPV1 modulators are most likely related to TRPV1 regulation by changes in [Ca^2+^]_i_ in TRCs that in turn modulates the phosphorylation state of the channel via activation of protein kinase C (PKC) and calcineurin [19]. In addition, changes in membrane phosphatidylinositol 4,5-bisphosphate (PIP_2_) in taste cells modulate the biphasic effects of RTX on the Bz-insensitive NaCl CT response [20]. Thus differences in the NGCC response of the expressed TRPV1 channel and the salt sensory perception in human subjects may reflect differences in the phosphorylation and regulation of the TRPV1/TRPV1t channel in the anterior taste receptive field.

In our studies αβγ hENaC was expressed in HEK293T cells and the activity of αβγ hENaC was confirmed by S3969. The EC_50_ value for S3969 in αβγ hENaC-expressing cells was 1.262±0.290 µM. This value is very close to the theoretical value (1.2±0.1 µM) obtained by patch-clamp [11]. Thus, αβγ hENaC-expressing cells were used to investigate the effect of NGCC on amiloride/benzamil-sensitive salt taste receptor. NGCC did not alter the membrane potential in αβγ hENaC-expressing cells, indicating that NGCC does not stimulate hENaC. This is consistent with our neural data in rodents in which Bz-sensitive component of the CT response remained unchanged after stimulating the tongue with salt solutions containing NGCC [27]. These results indicate that in both rodents and humans NGCC alters salt taste by specifically modulating only the Bz-insensitive component of the CT response in rodents. The NGCC-induced activation of hTRPV1 was confirmed by blocking hTRPV1 using RR or CPZ. These findings provide support for the role of NGCC in the activation of hTRPV1, as suggested previously [27]. Consistent with these *in vitro* studies, Bz-insensitive NaCl CT responses in the absence and presence of TRPV1 modulators were blocked by RR, CZP and SB-366719 in rats and WT mice [17], [18], [27], [34].

In addition, the effects of NGCC on ASIC was also evaluated because ASIC is amiloride-sensitive ion channel and a subfamily of the ENaC/Deg superfamily of ion channels. ASIC is expressed in mouse taste buds and proton-gated cation channel, therefore ASIC may be related to sour taste due to its activation by proton (H^+^) [31]. ASIC is activated by pH 5.9–6.5 of half maximal activation (pH_0.5_) and directly modulates PKA [35]. Although responding to protons, ASIC1-expressing cells were not activated by NGCC. That is, NGCC is not associated with sour taste via ASIC1-dependent pathway. In an earlier study [17], the Bz-insensitive CT response in rodents showed many properties that are similar to those observed with the cloned TRPV1 expressed in heterologous cells. The following common properties were observed between the Bz-insensitive NaCl CT responses and TRPV1: (i) activation by resiniferatoxin (RTX), capsaicinand elevated temperature; (ii) additive effects of temperature and vanilloids on the CT response; (iii) inhibition by TRPV1 blockers, RR, CZP and SB-366791; (iv) RTX produced biphasic changes in CT response to NaCl, KCl, NH_4_Cl and CaCl_2_ that were inhibited by SB-366791. This suggests that the Bz-insensitive CT responses to the above cations are dependent upon their influx through a non-selective cation channel; and (v) the absence of the constitutive Bz-insensitive NaCl CT response and insensitivity to vanilloids and temperature in TRPV1 KO mice. Moreover, spontaneous Bz-insensitive NaCl CT response was also observed at room temperature, in the absence of vallinoids and at the physiological pH. Finally, in the absence of vanilloids, the Bz-insensitive NaCl CT response was not affected by changes in the stimulus pH. These results led Lyall et al. to propose that the Bz-insensitive responses may be derived from a variant of TRPV1. Although TRPV1 and TRPV1t are not exactly identical, the experimental conditions used in this study (27°C, neutral pH, moderate ATP concentration) suggested that ligand-stimulated hTRPV1 activation could represent the activation of the putative amiloride-insensitive salt taste receptor.

TRPV1 is often co-expressed with the transient receptor potential channel ankyrin 1 (TRPA1), a member of the TRP channel family, in sensory nerve endings [36]. In sensory neurons, 97% of TRPA1-expressing cells co-express TRPV1, and 30% of TRPV1-expressing cells co-express TRPA1 [37]. Several compounds, such as 6-shogaol and 6-paradol, stimulate both TRPV1 and TRPA1 [38]. TRPA1 is a nonselective cation channel with high Ca^2+^ permeability [39]. Similar to TRPV1, TRPA1 is associated with somatosensation in response to environmental irritants, cold, and pain [40]. Using immunohistochemical studies, the expression of TRPA1 has been demonstrated in the human lingual trigeminal nerve and the nerve bundles of the mouse tongue [41], [42].

Treatment of hTRPA1-expressing cells with NGCC stimulated hTRPA1 and caused an increase in the intracellular Ca^2+^ concentration. The EC_50_ value for NGCC in hTRPA1-expressing cells was 83.65 µM. The response of hTRPA1-expressing cells to NGCC treatment was inhibited by RR and a specific hTRPA1 antagonist, HC-030031. It is likely that at high concentrations, the trigeminal effects of NGCC are due to its interactions with hTRPA1.

Interactions between hTRPA1 and salty tasting compounds have not been investigated. However, the similarity between hTRPA1 and hTRPV1 suggest that salty tasting compounds may interact with hTRPA1. hTRPA1 and hTRPV1 are both members of the TRP superfamily, Ca^2+^-permeable, and co-expressed in the same lingual nerve endings [43]. Human TRPA1 does not form a heterotetramer channel with hTRPV1; however, the formation of a complex between hTRPA1 and hTRPV1 may occur in the plasma membranes of sensory neurons. In addition, TRPV1 may influence the activity of the TRPA1 channel. In sensory neurons, capsaicin and mustard oil, a TRPA1 agonist, pharmacologically desensitized TRPA1 via Ca^2+^-dependent and independent pathways, respectively [44]. Both TRPA1 and TRPV1 channels could control transmission of inflammatory stimuli through nociceptors [45], [46]. Taken together, these results suggest that hTRPA1 channels may be activated by NGCC.

In our studies the constitutive Bz-insensitive NaCl CT response in the absence of TRPV1 modulators was not affected by changes extracellular pH between pH 2 and 10 [17]. Similarly, at constant external pH, changes in intracellular pH induced by exposing the TRCs in vivo to organic acids (e.g. CO_2_) did not alter the magnitude of the NaCl+Bz CT response. However, the NaCl+Bz CT response in the presence of several TRPV1 agonists (RTX, ethanol, nicotine, and MRPs) varied as a function of extracellular pH. The relationship between pH and the magnitude of the CT response was bell shaped. The maximum increase in the CT response was observed at a pH between 6.0 and 6.5 [17], [18], [34]. TRPA1 is activated by changes in intracellular pH only [47]. This suggests that TRPA1 is most likely not involved in the Bz-insensitive response in the absence and presence of TRPV1 modulators. Rather these data suggest that a variant (TRPV1t) rather than TRPV1 is involved in Bz-insensitive salt responses [17].

In conclusion, the results presented in this paper indicate that NGCC modulates the activity of hTRPV1 and hTRPA1 in a concentration dependent manner. The interaction between NGCC and hTRPV1 are most likely related to the observed alteration in the salty taste in humans in the presence of NGCC. The interactions of NGCC with TRPA1 most likely contribute to its trigeminal effects at high concentrations. This study suggested that NGCC or related compounds can be used to reduce salt consumption, which could prevent the adverse effects of excessive salt consumption, such as hypertension, heart attack, and stroke.
